# Supplementary figures and images for: Emerging cooperativity between Oct4 and Sox2 governs the pluripotency network in early mouse embryos
Source: eLife. 2025 Feb 27;13:RP100735. doi: 10.7554/eLife.100735 (PMC11867617; doi:10.7554/eLife.100735)

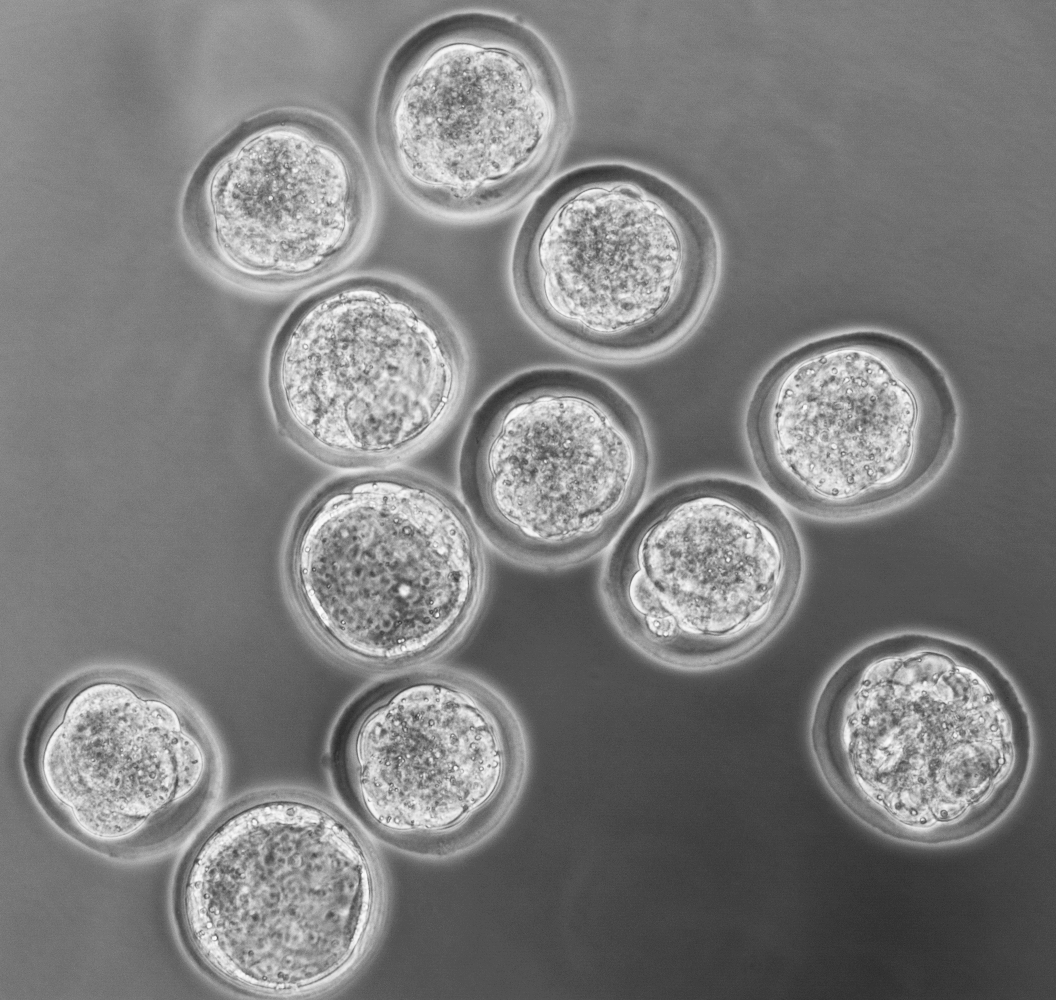

Supplement: Figure 1—figure supplement 1—source data 1. [file elife-100735-fig1-figsupp1-data1.zip › Figure 1-source data 1/Figure 1-figure supplement 1A_Pou5f1/mKO2+_E2,5+1,5_ch00.png]

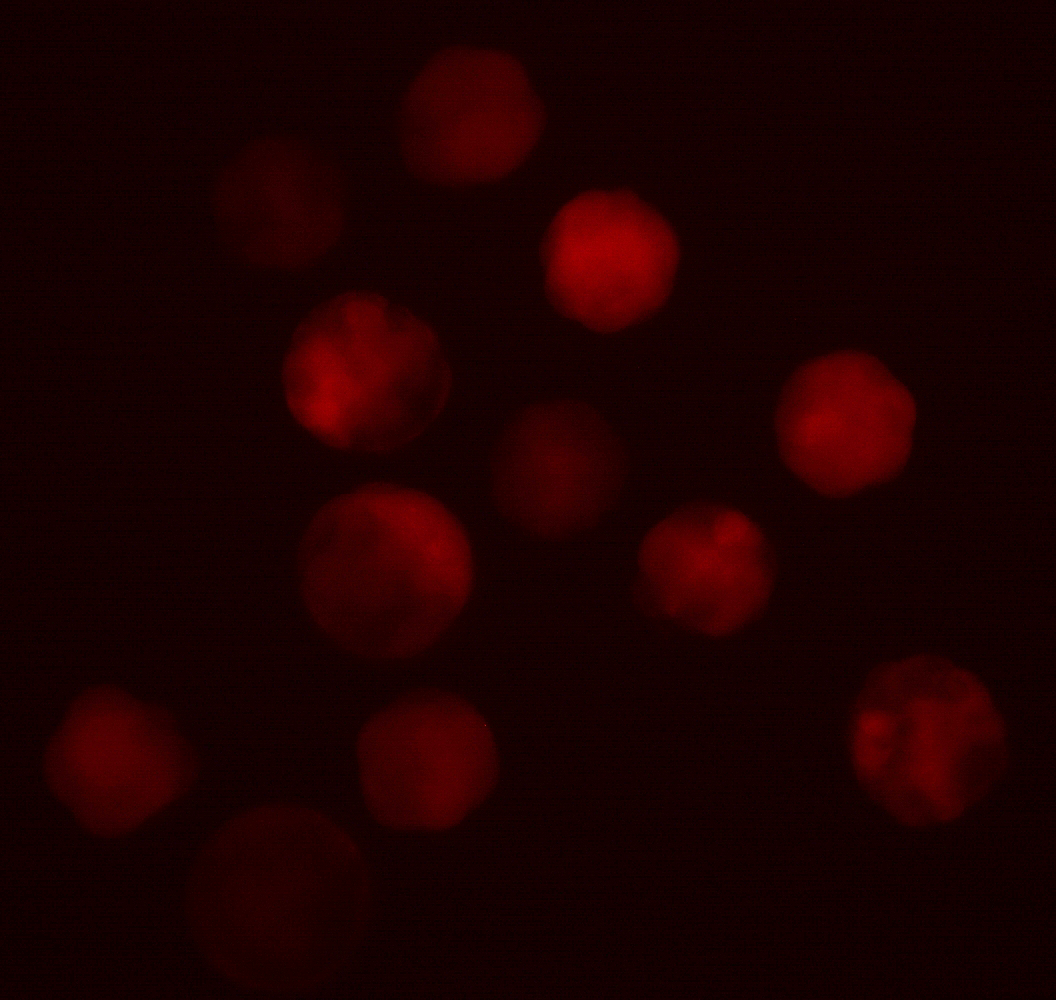

Supplement: Figure 1—figure supplement 1—source data 1. [file elife-100735-fig1-figsupp1-data1.zip › Figure 1-source data 1/Figure 1-figure supplement 1A_Pou5f1/mKO2+_E2,5+1,5_ch01.png]

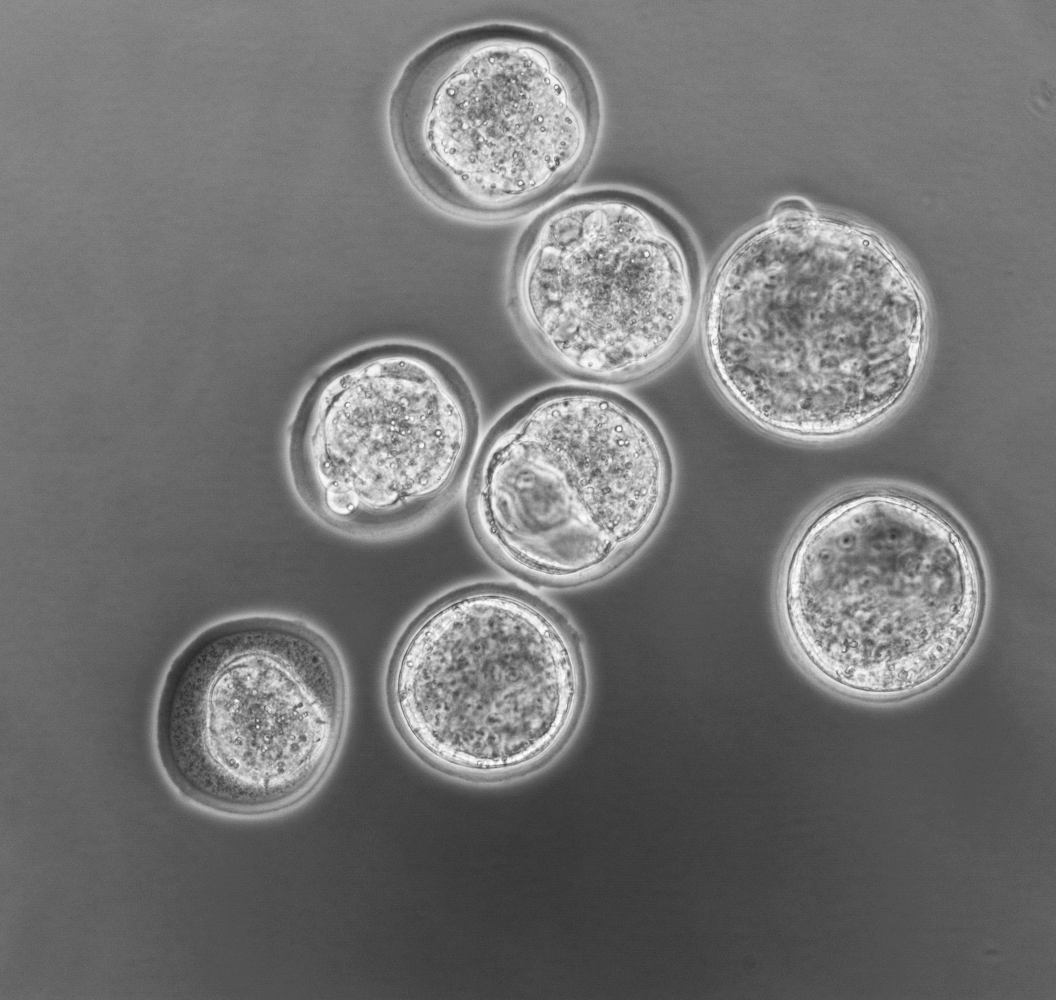

Supplement: Figure 1—figure supplement 1—source data 1. [file elife-100735-fig1-figsupp1-data1.zip › Figure 1-source data 1/Figure 1-figure supplement 1A_Pou5f1/mKO2-_E2,5+1,5_ch00.png]

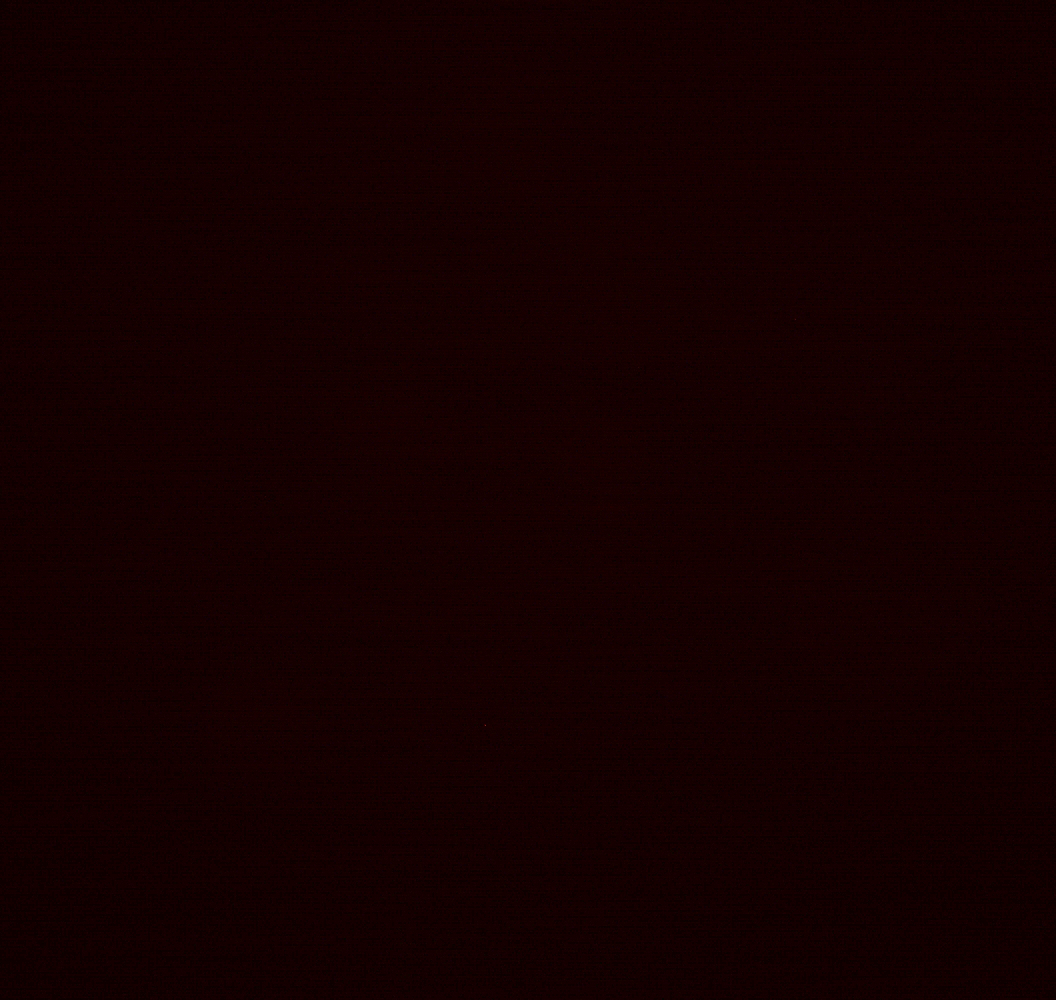

Supplement: Figure 1—figure supplement 1—source data 1. [file elife-100735-fig1-figsupp1-data1.zip › Figure 1-source data 1/Figure 1-figure supplement 1A_Pou5f1/mKO2-_E2,5+1,5_ch01.png]

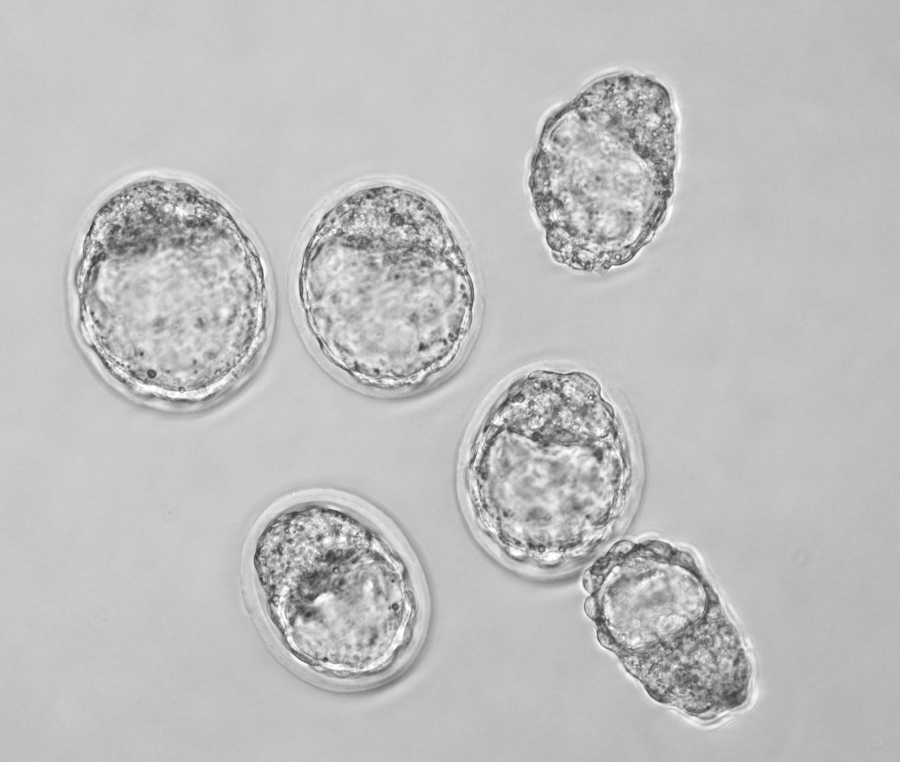

Supplement: Figure 1—figure supplement 1—source data 1. [file elife-100735-fig1-figsupp1-data1.zip › Figure 1-source data 1/Figure 1-figure supplement 1B_Sox2/EGFP+_ch00.png]

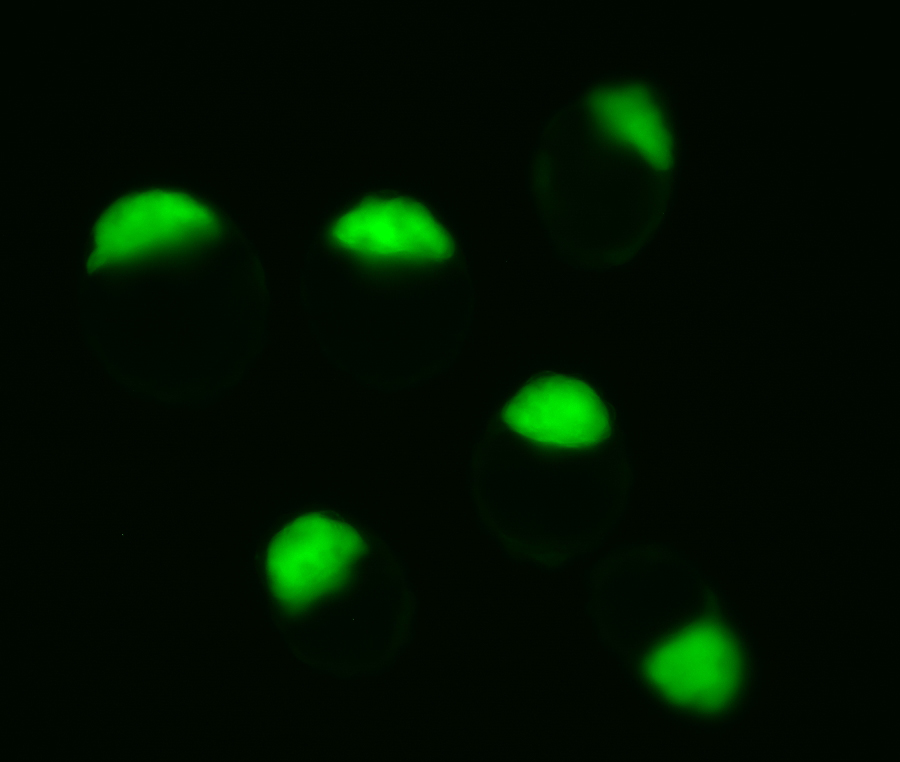

Supplement: Figure 1—figure supplement 1—source data 1. [file elife-100735-fig1-figsupp1-data1.zip › Figure 1-source data 1/Figure 1-figure supplement 1B_Sox2/EGFP+_ch01.jpg]

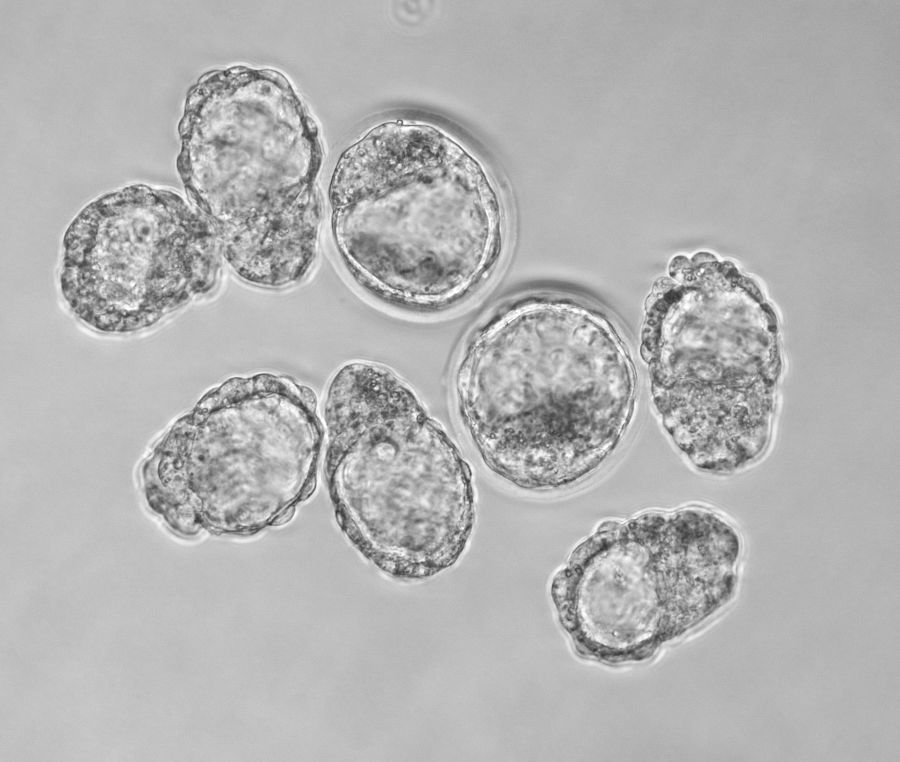

Supplement: Figure 1—figure supplement 1—source data 1. [file elife-100735-fig1-figsupp1-data1.zip › Figure 1-source data 1/Figure 1-figure supplement 1B_Sox2/EGFP-_ch00.png]

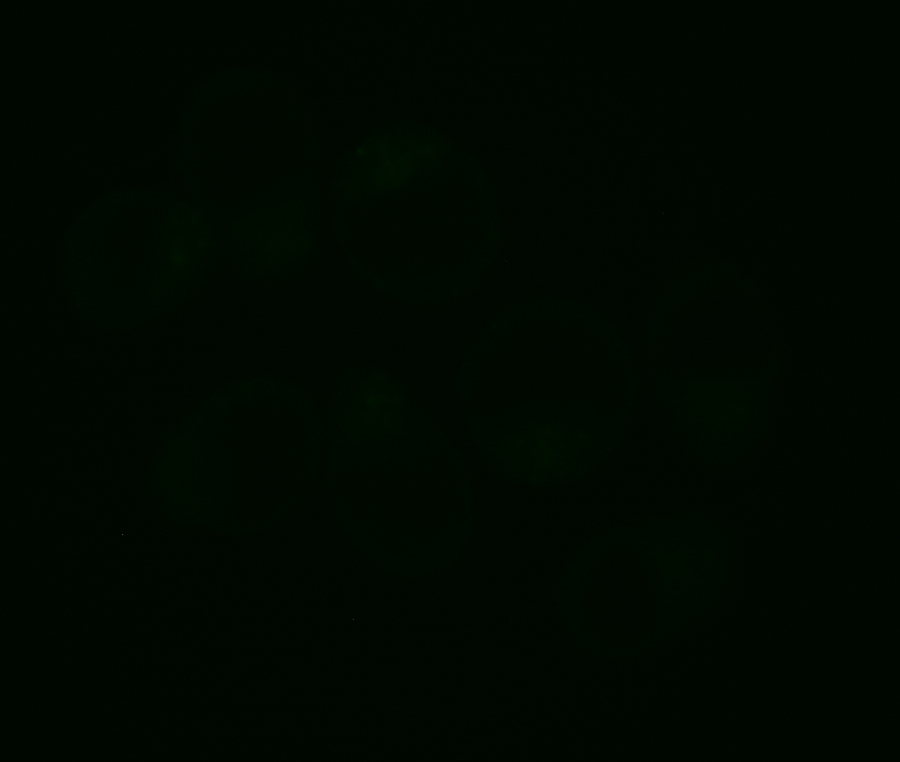

Supplement: Figure 1—figure supplement 1—source data 1. [file elife-100735-fig1-figsupp1-data1.zip › Figure 1-source data 1/Figure 1-figure supplement 1B_Sox2/EGFP-_ch01.jpg]
